# Supplementary material for: Content-rich biological network constructed by mining PubMed abstracts
Source: BMC Bioinformatics. 2004 Oct 8;5:147. doi: 10.1186/1471-2105-5-147 (PMC528731; doi:10.1186/1471-2105-5-147)
Supplement: Additional File 2 — The original results of the above study (non-essential files are deleted to keep the file size under the limit set by BMC bioinformatics). [file 1471-2105-5-147-S2.bz2 › chilibotAdditionalFile2/dip10/248ID8776728E824/html/HSPD1_HSPA1A.html]

 


 **HSPD1** and **HSPA1A** 
  
Found 8 abstracts in PubMed, retrieved 8.  
 

 What does Google say? 
 PDF only 
| .edu only 

---

**Interactive relationship** (e.g. stimulation, inhibition, etc)

**Non-interactive relationship** (e.g. studied together, co-existance, homology, etc.)

- As in any other bacterium, Helicobacter pylori synthesizes two heat shock proteins, the HspA  [ **HSPA1A** ]  GroES or Hsp 10 homologue and the HspB GroEL  [ **HSPD1** ]  or Hsp60  [ **HSPD1** ]  homologue.  Ref: 8730259 Aliment Pharmacol Ther, 1996
- We investigated whether Helicobacter pylori cells actively secrete proteins such as the urease subunits UreA and UreB and the GroES and GroEL  [ **HSPD1** ]  homologs HspA  [ **HSPA1A** ]  and HspB or whether these proteins were present in the extracellular compartment as a consequence of autolysis.  Ref: 9488391 Infect Immun, 1998
- The gene encoding the GroEL  [ **HSPD1** ]  like protein of H. pylori HspB was cloned pILL689 and was shown to belong to a bicistronic operon including the hspA  [ **HSPA1A** ]  and hspB genes.  Ref: 7715457 Mol Microbiol, 1994
- We have recently shown that the N terminal ATPase fragment of hsp70 1  [ **HSPA1A** ]  375, composed of domains I and II as well as the subsequent domain III 376 520 may share three dimensional similarities with hsp60  [ **HSPD1** ] .  Ref: 7882155 C R Acad Sci III, 1994
- Neither fruiting body formation nor sporulation, nor the production of possible SigB targets, such as DnaK, GroEL  [ **HSPD1** ]  or HspA  [ **HSPA1A** ] , were affected.  Ref: 11496003 Microbiology, 2001
- In growth above pH 7, three stress proteins were induced GroES HspA  [ **HSPA1A** ] , GroEL  [ **HSPD1** ]  HspB, and the antioxidant AhpC homolog TsaA.  Ref: 11179990 Helicobacter, 2000
- METHODS Helicobacter pylori multiplex PCR and RT PCR assays were carried out using primer pairs targeting the cagA, vacA, ureA, ureI, hspA  [ **HSPA1A** ]  hsp60  [ **HSPD1** ] , and sodB genes and their cDNA.  Ref: 12390208 Helicobacter, 2002
